# MiR-770-5p facilitates podocyte apoptosis and inflammation in diabetic nephropathy by targeting TIMP3

**DOI:** 10.1042/BSR20193653

**Published:** 2020-04-29

**Authors:** Li Wang, Hua Li

**Affiliations:** 1 Department of Geriatrics, Xiangyang NO. 1 People's Hospital, Hubei University of Medcine, Xiangyang, Hubei, China; 2 Department of Emergency, Xiangyang NO. 1 People's Hospital, Hubei University of Medcine, Xiangyang, Hubei, China

**Keywords:** diabetic nephropathy, inflammation, miR-770-5p, podocyte apoptosis, TIMP3

## Abstract

Objective: Diabetic nephropathy (DN) is one of the most severe and frequent diabetic complications. MicroRNAs (miRNAs) have been reported to play a vital role in DN pathogenesis. The present study aimed to investigate the molecular mechanism of miR-770-5p in DN.

Methods: Podocyte injury model was established by treating mouse podocytes with high glucose (HG, 33 mM) for 24 h. The levels of miR-770-5p and TIMP3 were examined in kidney tissues and podocytes using quantitative real-time PCR (qRT-PCR). Flow cytometry analysis was applied to detect apoptosis in podocytes. Western blot assay was used to measure the protein levels of B-cell lymphoma 2 (Bcl-2), Bcl-2 associated X (Bax) and tissue inhibitors of metalloproteinase 3 (TIMP3). Enzyme-linked immunosorbent assay (ELISA) was conducted to measure the levels of inflammatory factors. The interaction between miR-770-5p and TIMP3 was determined by MicroT-CDS and luciferase reporter assay.

Results: MiR-770-5p was up-regulated and TIMP3 was down-regulated in DN kidney tissues and HG-stimulated podocytes. Depletion of miR-770-5p suppressed cell apoptosis and the release of pro-inflammatory factors in HG-treated podocytes. Additionally, TIMP3 was a target of miR-770-5p in HG-treated podocytes. TIMP3 inhibited cell apoptosis and inflammation in HG-treated podocytes. Moreover, TIMP3 knockdown alleviated the inhibitory effect of miR-770-5p silencing on podocyte apoptosis and inflammatory response.

Conclusion: Knockdown of miR-770-5p suppressed podocyte apoptosis and inflammatory response by targeting TIMP3 in HG-treated podocytes, indicating that miR-770-5p may be a potential therapeutic target for DN therapy.

## Introduction

Diabetic nephropathy (DN) is a microvascular complication of diabetes and the leading cause of end-stage renal disease [1]. The typical hallmark of DN is the precipitation of extracellular matrix (ECM) in mesangium and renal tubulo-interstitium, and the thickening of glomerular and tubular basement membranes, resulting in glomerulosclerosis and tubulo-interstitial fibrosis [2]. Although some progress has been made in DN treatment, the pathogenesis of DN still needs to be further explored to discover new biomarkers and therapeutic targets.

Podocytes are a class of highly differentiated postmitotic cells involved in the glomerular filtration barrier [3]. Podocyte injury is considered to be an early pathological mechanism of various glomerular diseases, leading to glomerular filtration barrier dysfunction and proteinuria [4]. Increasing evidence demonstrated that apoptosis and autophagy play crucial roles in DN pathogenesis [5]. Programmed cell death is determined by the interaction between apoptosis-inhibiting and apoptosis-promoting protein in the B-cell lymphoma 2 (Bcl-2) protein family [6]. The apoptosis inhibitory protein Bcl-2 prevents apoptosis, while apoptosis-inducing protein Bcl-2 Associated X (Bax) induces the release of caspase [7,8]. In addition, inflammation directly destroys renal structure and is closely related to the progression of DN [9].

MicroRNAs (miRNAs) are highly conserved short noncoding RNAs, 18–25 nucleotides in length, and are the major post-transcriptional regulators of gene expression [10]. Recently, more and more studies have implicated that miRNAs occupy a vital position in pathophysiology of renal diseases [11,12]. Moreover, the effects of some miRNAs on DN pathophysiology have been identified, revealing that miRNAs are promising therapeutic targets in DN [13]. For example, miR-30c hindered epithelial-to-mesenchymal transition (EMT) to prevent diabetic nephropathy by regulating Snail1–TGF-β1 pathway [14]. Silencing of miR-21 suppressed inflammation and podocyte apoptosis in diabetic nephropathy via targeting tissue inhibitor of metalloproteinase-3 (TIMP3) [15]. Furthermore, miR-770-5p was up-regulated in high glucose-induced podocytes, and knockdown of miR-770-5p suppressed high glucose-triggered podocyte apoptosis by targeting TP53 and regulating inhibitor of apoptosis 1 (TRIAP1) [16]. However, the effect of miR-770-5p on high glucose-induced inflammation remains largely unknown.

Moreover, the loss of TIMP3 exacerbates the progress of DN [17]. TIMP3 is an essential mediator of kidney injury, and the lack of TIMP3 increases interstitial nephritis and fibrosis as reported previously [18]. However, the relationship between TIMP3 and miR-770-5p on DN development is unclear.

In the present study, we used HG-treated podocyte injury model to investigate the role of miR-770-5p in diabetic nephropathy pathogenesis.

## Materials and methods

### Tissue specimens

The kidney tissues were acquired from 25 DN patients and 25 age-matched healthy volunteers from Xiangyang No. 1 People’s Hospital Affiliated to Hospital of Hubei University of Medicine. Patients with DN did not receive any therapy prior to enrolling in this subject. The research was ratified by the Ethics Committee of Xiangyang No. 1 People’s Hospital Affiliated to Hospital of Hubei University of Medicine. Written informed consent was signed by all participants.

### Cell culture

Mouse podocytes (Yiyan, Shanghai, China) were seeded into RPMI-1640 medium (Gibco, Carlsbad, CA, U.S.A.) supplemented with 10% fetal bovine serum (Gibco) and 0.5% penicillin/streptomycin at 33°C for proliferation. Then, podocytes were incubated at 37°C for differentiation. For high-glucose treatment, podocytes were incubated in serum-free medium for 12 h, and then were treated with 33 mM high glucose (HG) for indicated time. About 5.5 mM glucose (NC) and 5.5 mM glucose + 27.5 mM mannitol (Mannitol) were used as controls.

### Cell transfection

When cell confluence reached ∼70%, cell transfection was carried out using Lipofectamine 2000 (Invitrogen, Carlsbad, CA, U.S.A.). MiR-770-5p inhibitor (in-miR-770-5p#1, in-miR-770-5p#2 and in-miR-770-5p#3), the negative control inhibitor (in-miR-NC), miR-770-5p mimic (miR-770-5p), the control mimic (miR-NC), TIMP3 overexpression vector (TIMP3), the empty vector (pcDNA), small interfering RNA (siRNA) of TIMP3 (si-TIMP3) and the control siRNA (si-NC) were synthesized by GenePharma (Shanghai, China).

### Quantitative real-time PCR (qRT-PCR)

In renal tissues and podocytes, RNA was isolated using Trizol reagent (Invitrogen). RNA was reverse transcribed into cDNA using miScript Reverse Transcription Kit (Qiagen, Frankfurt, Germany) or HiScript II One Step RT-PCR Kit (Vazyme, Nanjing, China). The expression was detected using SYBR Green PCR Master Mix (Thermo Fisher Scientific, Waltham, MA, U.S.A.). The gene expression was calculated by 2^−ΔΔCt^. Primers were exhibited below: miR-770-5p (forward, 5′-AGCACCACGTGTCTGG-3′; reverse, 5′-GAACATGTCTGCGTATCTC-3′), TIMP3 (forward, 5′-CTTCTGCAACTCCGACATCGT-3′; reverse, 5′-GGGGCATCTTACTGAAGCCTC-3′), U6 (forward, 5′-GCTTCGGCAGCACATATACTAAAAT-3′; reverse, 5′-CGCTTCACGAATTTGCGTGTCAT-3′), β-actin (forward, 5′-GTGACGTTGACATCCGTAAAGA-3′; reverse, 5′-GCCGGACTCATCGTACTCC-3′). U6 or β-actin was used as internal control.

### Apoptosis analysis

Cell apoptotic rate was estimated using Annexin V, FITC Apoptosis Detection Kit (Dojindo, Kumamoto, Japan). Briefly, podocytes were seeded into six-well plates and cleaned with phosphate-buffered saline (PBS). Next, cells were stained with annexin V-FITC and propidium iodide (PI) for 30 min. The apoptotic rate was monitored using Attune NxT Flow Cytometer (Thermo Fisher Scientific).

### Western blot assay

Total protein was lysed using RIPA buffer (Invitrogen). Equal amounts of protein samples were separated by sodium dodecyl sulfate polyacrylamide gel electrophoresis (SDS-PAGE) and transferred to polyvinylidene fluoride membranes (Millipore, Billerica, MA, U.S.A.). After blocking in 5% skim milk for 2 h, membranes were incubated with primary antibodies against Bcl-2, Bax, TIMP3 or β-actin (1:1,000; Abcam, Cambridge, U.K.) at 4°C overnight and incubated with corresponding secondary antibody (1:4000; Abcam) for 2 h at room temperature. The protein bands were visualized by enhanced chemiluminescence reagents (Millipore).

### Enzyme-linked immunosorbent assay (ELISA)

Podocytes were seeded in 24-well plates and treated with different conditions. Then, the levels of interleukin 1β (IL-1β) and tumor necrosis factor-α (TNF-α) were detected using ELISA kits (RD, Minneapolis, MN, U.S.A.).

### Luciferase reporter assay

The fragments of TIMP3 3′ UTR containing the wild-type binding sites of miR-770-5p (TIMP3-3′ UTR-WT) or the mutant (TIMP3-3′ UTR-MUT) were inserted into pGL3 plasmids (Promega, Madison, WI, U.S.A.). Then, recombinant vectors and miR-NC, miR-770-5p, in-miR-NC or in-miR-770-5p were co-transfected into podocytes with Lipofectamine 2000 (Invitrogen). Luciferase activity was analyzed using Dual-Luciferase Reporter Assay System (Promega).

### Statistical analysis

Data were displayed as mean ± standard deviation from three independent experiments. Significance was calculated by Student’s *t*-test or one-way analysis of variance (ANOVA). Data were analyzed using Graphpad Prism 7.0 software (GraphPad, San Diego, CA, U.S.A.). Differences were considered statistically significant at *P*<0.05.

## Results

### MiR-770-5p was up-regulated and TIMP3 was down-regulated in DN kidney tissues and HG-induced podocytes

**Figure 1:**
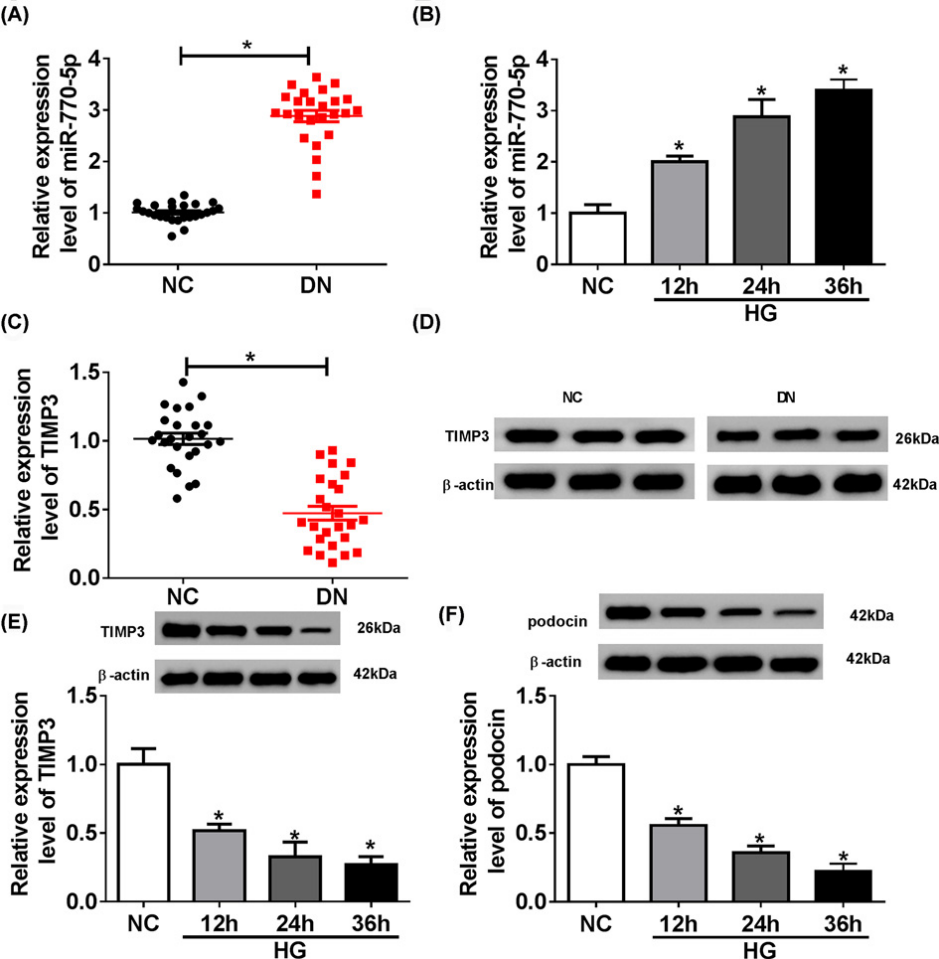
Expression levels of miR-770-5p and TIMP3 in DN kidney tissues and HG-induced podocytes (**A** and **B**) The expression level of miR-770-5p was measured in DN kidney tissues and HG-induced podocytes and their control tissues and podocytes by qRT-PCR. (**C–E**) The expression level of TIMP3 was examined in DN kidney tissues and HG-induced podocytes and their control tissues and podocytes by qRT-PCR and Western blot. (**F**) The protein level of podocin was detected in podocytes treated with high glucose at different times by Western blot; *P<0.05.

First, to investigate the effects of miR-770-5p and TIMP3 on DN development, the levels of miR-770-5p and TIMP3 were examined in renal tissues of DN patients and healthy volunteers. The results of qRT-PCR and Western blot suggested that miR-770-5p expression was remarkably increased, while TIMP3 expression was dramatically decreased in renal tissues of DN patients compared with the negative control (NC) group (Figure 1A,C,D). Next, podocytes were incubated in 33 mM glucose for 12, 24 and 36 h, respectively. Then, qRT-PCR and Western blot exhibited that miR-770-5p level was apparently elevated in a time-dependent manner, while TIMP3 and podocin levels were strikingly reduced in a time-dependent manner in HG-treated podocytes (Figure 1B,E,F). As shown in Supplementary Figure S1, Western blot assay showed that the levels of nephrin, pododin and WT1 were significantly reduced in HG-treated podocytes relative to the negative control group. As shown in Supplementary Figure S2, miR-770-5p expression was increased, while TIMP3 expression was decreased in the HG group compared with the NC and Mannitol groups.

### Depletion of miR-770-5p alleviated HG-induced apoptosis and inflammation in HG-induced podocytes

**Figure 2:**
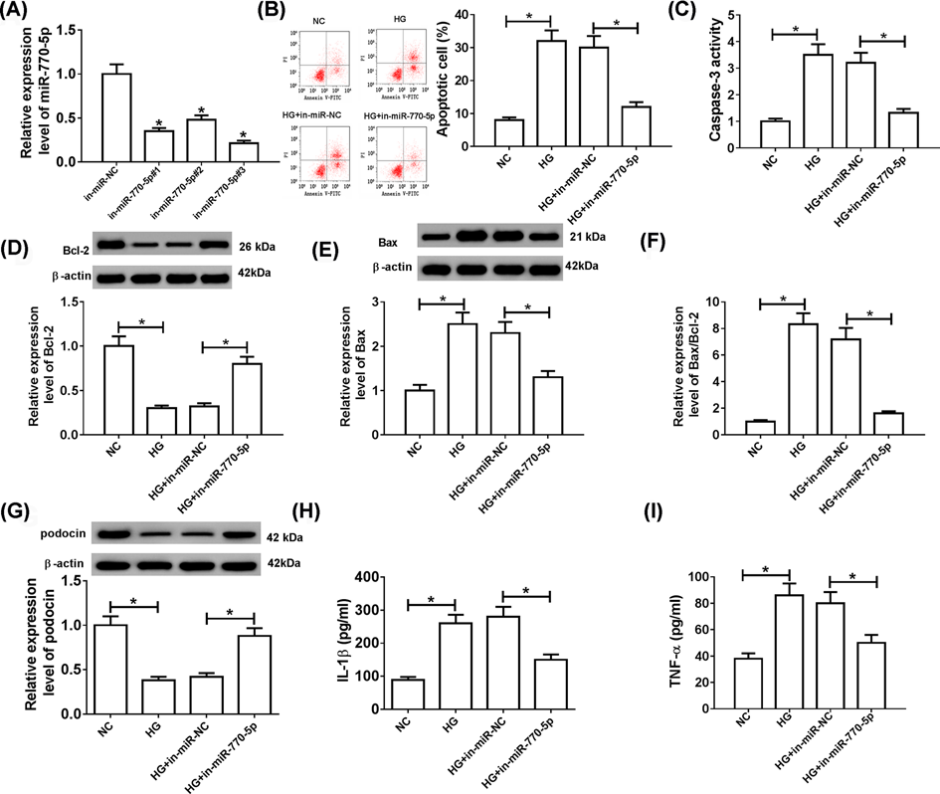
Depletion of miR-770-5p alleviated HG-induced apoptosis and inflammation in podocytes HG-treated podocytes were transfected with in-miR-NC, in-miR-770-5p#1, in-miR-770-5p#2 or in-miR-770-5p#3. (**A**) The expression level of miR-770-5p was detected by qRT-PCR analysis. (**B**) Cell apoptotic rate was evaluated by flow cytometry. (**C–F**) Western blot analysis was performed to measure Caspase-3 activity as well as Bcl-2 and Bax levels. (**G**) The protein level of podocin was measured using Western blot assay. (**H** and **I**) The levels of inflammatory cytokines (IL-1β and TNF-α) were detected using ELISA analysis; *P<0.05.

In order to evaluate the role of miR-770-5p in the progression of DN, HG-induced podocytes were transfected with in-miR-770-5p#1, in-miR-770-5p#2 or in-miR-770-5p#3 to down-regulate the expression of miR-770-5p. The results of qRT-PCR revealed that miR-770-5p inhibitors decreased miR-770-5p expression compared with in-miR-NC group, and in-miR-770-5p#3 (in-miR-770-5p) had the highest knockdown efficiency and was used for subsequent experiments (Figure 2A). Moreover, HG-stimulated podocytes were transfected with in-miR-NC or in-miR-770-5p. Then, flow cytometry suggested that the apoptosis rate of podocytes was evidently increased after HG treatment, but the effect was abolished after transfection with in-miR-770-5p (Figure 2B). Consistently, Bcl-2 protein level was distinctly decreased, while Caspase-3 activity, Bax level and the ratio of Bax/Bcl-2 were prominently increased in HG-treated podocytes compared with the negative control group, and these effects were reversed after transfection with in-miR-770-5p (Figure 2C–F). Also, inhibition of miR-770-5p reversed the decline in podocin expression caused by HG stimulation (Figure 2G). Furthermore, HG treatment facilitated the release of IL-1β and TNF-α in the supernatant of podocytes medium, whereas the effect was abrogated by inhibiting miR-770-5p (Figure 2H,I). These data indicated that knockdown of miR-770-5p alleviated HG-induced apoptosis and inflammation in podocytes.

### TIMP3 was a target of miR-770-5p

**Figure 3:**
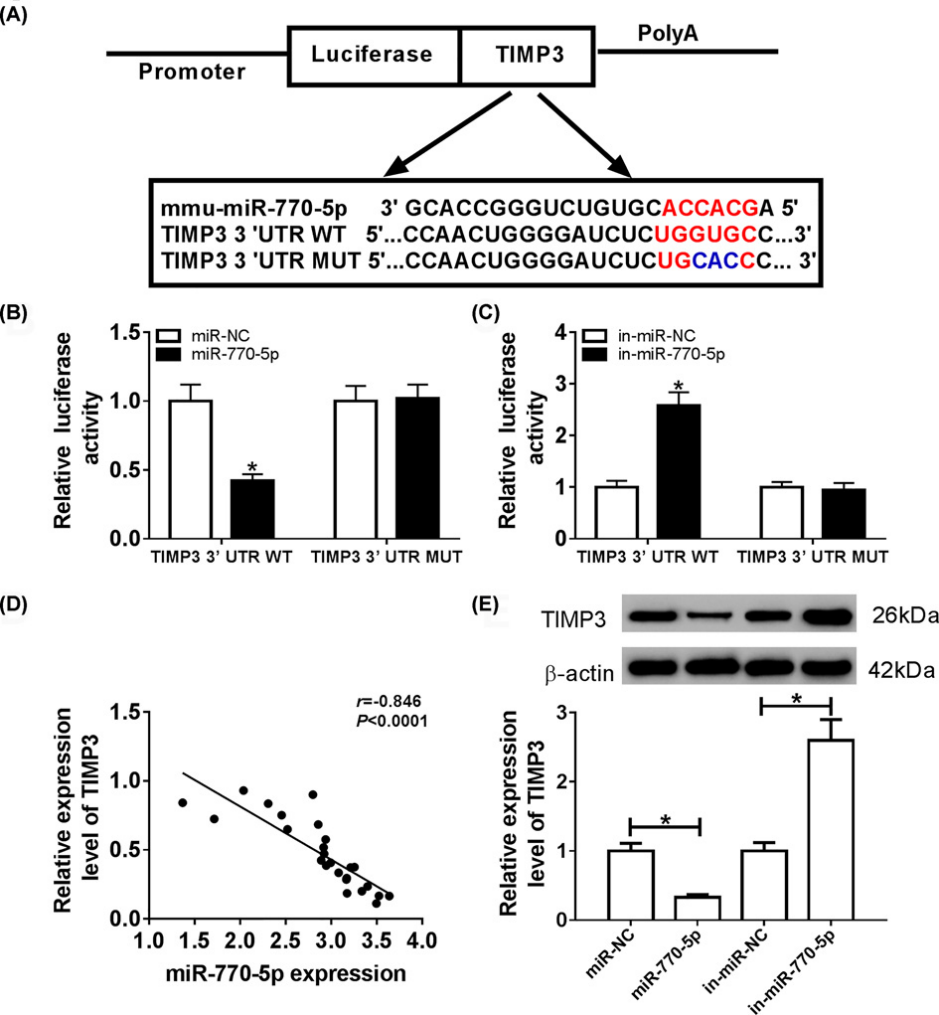
TIMP3 was a target of miR-770-5p (**A**) The predicted binding sites between miR-770-5p and 3′UTR of TIMP3 were shown. (**B**) Luciferase reporter assay was performed to analyze the luciferase activity in podocytes co-transfected with TIMP3-3′UTR-WT or TIMP3-3′UTR-MUT and miR-NC or miR-770-5p mimic. (**C**) Luciferase activity was detected in podocytes co-transfected with TIMP3-3′UTR-WT or TIMP3-3′UTR-MUT and in-miR-NC or miR-770-5p inhibitor. (**D**) The correlation between miR-770-5p and TIMP3 in renal tissues of DN patients was analyzed. (**E**) Western blot assay was applied to measure the protein level of TIMP3 in HG-induced podocytes transfected with miR-NC, miR-770-5p, in-miR-NC or in-miR-770-5p, respectively; *P<0.05.

To investigate the underlying molecular mechanism of miR-770-5p in DN, MicroT-CDS online database was used to predict the putative targets of miR-770-5p. TIMP3 3′UTR contained the binding sites of miR-770-5p (Figure 3A). Luciferase reporter assay demonstrated that mature miR-770-5p markedly restrained the luciferase activity of TIMP3-3′UTR-WT reporter in podocytes, but did not affect the luciferase activity of TIMP3-3′UTR-MUT reporter (Figure 3B). In addition, miR-770-5p inhibitor apparently elevated the luciferase activity of TIMP3-3′UTR-WT reporter, while the luciferase activity was not affected when the binding sites were mutated (Figure 3C). Moreover, the expression levels of miR-770-5p and TIMP3 were negatively correlated in renal tissues of patients with DN (Figure 3D). The protein level of TIMP3 was detected in podocytes transfected with miR-NC, miR-770-5p, in-miR-NC or in-miR-770-5p, respectively. The results of Western blot analysis exhibited that miR-770-5p mimic conspicuously down-regulated the protein level of TIMP3, and miR-770-5p inhibitor remarkably up-regulated the protein level of TIMP3 (Figure 3E). All these data demonstrated that miR-770-5p directly targeted TIMP3 and negatively regulated TIMP3 in podocytes.

### TIMP3 inhibited apoptosis and inflammation in HG-treated podocytes

**Figure 4:**
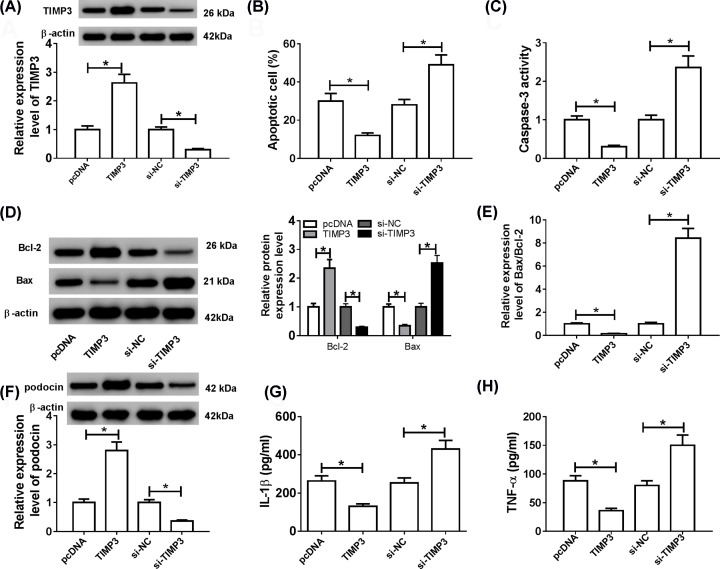
TIMP3 inhibited apoptosis and inflammation in HG-treated podocytes HG-stimulated podocytes were transfected with pcDNA, TIMP3, si-NC or si-TIMP3, respectively. (**A**) The protein level of TIMP3 was examined using Western blot. (**B**) Cell apoptotic rate was estimated by flow cytometry. (**C**–**E**) Caspase-3 activity, Bcl-2 and Bax levels, and Bax/Bcl-2 ratio were detected using Western blot analysis. (**F**) The expression of podocin was examined by Western blot assay. (**G** and** H**) The levels of inflammatory cytokines (IL-1β and TNF-α) were measured by ELISA analysis; *P<0.05.

To explore the effects of TIMP3 on the progression of DN, HG-stimulated podocytes were transfected with pcDNA, TIMP3, si-NC or si-TIMP3, respectively. First, overexpression of TIMP3 led to an apparent increase of TIMP3 protein expression, and suppression of TIMP3 led to an evident decrease of TIMP3 protein expression in HG-induced podocytes (Figure 4A). Flow cytometry analysis exhibited that the apoptosis rate was remarkably reduced in with TIMP3 group compared with the pcDNA group, and the apoptosis rate was markedly enhanced in si-TIMP3 group compared with the si-NC group (Figure 4B). Similarly, TIMP3 overexpression increased Bcl-2 protein level and reduced Caspase-3 activity, Bax expression and Bax/Bcl-2 ratio, while down-regulation of TIMP3 had the opposite effect (Figure 4C–E). TIMP3 overexpression overtly increased podocin level, while TIMP3 silencing strikingly reduced podocin level (Figure 4F). In addition, up-regulation of TIMP3 significantly inhibited the release of IL-1β and TNF-α, and TIMP3 silencing dramatically promoted the release of IL-1β and TNF-α (Figure 4G,H). These data indicated that TIMP3 suppressed apoptosis and inflammation in HG-treated podocytes.

### TIMP3 knockdown abrogated the inhibitory effects on apoptosis and inflammation in HG-stimulated podocytes mediated by miR-770-5p inhibitor

**Figure 5:**
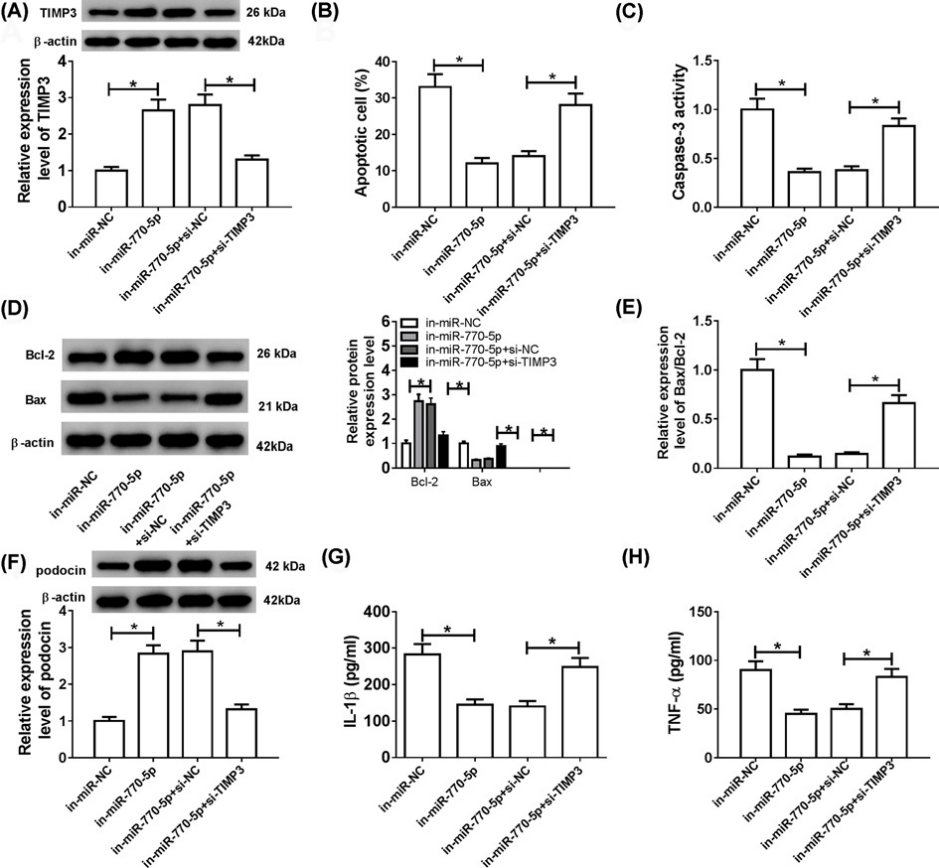
TIMP3 knockdown abrogated the inhibitory effects of miR-770-5p knockdown on apoptosis and inflammation in HG-stimulated podocytes HG-induced podocytes were transfected with in-miR-NC, in-miR-770-5p, in-miR-770-5p+si-NC or in-miR-770-5p+si-TIMP3, respectively. (**A**) Western blot assay was used to detect the protein level of TIMP3. (**B**) Flow cytometry was conducted to monitor cell apoptosis. (**C–E**) Caspase-3 activity, Bcl-2 and Bax levels, and Bax/Bcl-2 ratio were examined using western blot assay. (**F**) The expression of podocyte-specific marker (podocin) was detected by western blot assay. (**G** and **H**) The levels of inflammatory cytokines (IL-1β and TNF-α) were measured by ELISA analysis; *P<0.05.

To further investigate the roles of miR-770-5p and TIMP3 in DN development, HG-treated podocytes were transfected with in-miR-NC, in-miR-770-5p, in-miR-770-5p+si-NC or in-miR-770-5p+si-TIMP3. The results revealed that inhibition of miR-770-5p resulted in an evident increase of TIMP3 protein expression, while the effect was abolished after suppression of TIMP3 (Figure 5A). Additionally, knockdown of miR-770-5p conspicuously decreased the apoptosis rate of podocytes, whereas this effect was reverted by down-regulating TIMP3 (Figure 5B). Meanwhile, knockdown of miR-770-5p elevated Bcl-2 expression and reduced Caspase-3 activity, Bax expression and Bax/Bcl-2 ratio, but these impacts were restored after transfection with si-TIMP3 (Figure 5C–E). In addition, transfection with si-TIMP3 attenuated the increase in podocin level caused by miR-770-5p inhibition (Figure 5F). ELISA analysis showed that the levels of IL-1β and TNF-α were notably decreased in in-miR-770-5p group compared with in-miR-NC group, while the levels of inflammation factors were recuperated after transfection with TIMP3 interference (Figure 5G,H). All these data reflected that TIMP3 knockdown overturned the constraint impacts on apoptosis and inflammation in HG-stimulated podocytes caused by down-regulation of miR-770-5p.

## Discussion

Although DN pathogenesis has made progress, its high incidence and poor prognosis have not been greatly improved [19]. Therefore, excavating novel therapeutic targets for DN is significant. The pathogenesis of DN includes the release of pro-inflammatory cytokines [20]. Podocyte hypertrophy, podocyte detachment and podocyte apoptosis may lead to the increase of urinary protein in DN [21]. In addition, podocyte injury occupies a crucial position in DN pathogenesis [22]. Podocin is a podocyte-specific marker, and the loss of podocyte-specific markers may lead to podocyte injury [23]. Moreover, up-regulated transforming growth factor-β (TGF-β) accelerates the production of extracellular matrix proteins in renal cells, which leads to glomerulosclerosis and tubulointerstitial fibrosis [24]. Also, the mechanical stretch that causes loss of nephrin and α3β1 integrin is a factor in podocyte apoptosis and detachment [25]. Therefore, in order to improve the present study, TGF-β and mechanical stretch in podocytes need to be measured in future studies. In the present study, HG-stimulated podocytes were used to mimic podocyte injury to study the pathogenesis of DN.

Increasing evidence revealed that miRNAs occupy a vital position in the pathophysiology of renal diseases [26,27]. For instance, miR-15b-5p alleviated podocyte injury triggered by HG via regulating Sema3A expression [28]. MicroRNA-195 facilitated apoptosis of podocytes treated with high-glucose through elevating caspase activity for BCL2 insufficiency [29]. MiR-874 relieved podocyte injury in DN via regulating TLR4 expression [30]. MiR-320a facilitated DN development via targeting MafB and relieving Nephrin and Gpx3 [31]. MiR-770-5p expression was enhanced in HG-treated podocytes, and silencing of miR-770-5p relieved HG-induced podocyte apoptosis via targeting TRIAP1 [16]. Furthermore, miR-770-5p strengthened the radiosensitivity of tumors by negatively regulating PBK [32]. Additionally, miR-770-5p targeted HIPK1 to regulate the resistance of colon cancer to methotrexate [33]. Consistent with previous reports, miR-770-5p was distinctly up-regulated in DN. Knockdown of miR-770-5p alleviated apoptosis and inflammation in HG-triggered podocytes.

Furtherly, to investigate the underlying mechanism of miR-770-5p in DN progression, the targets of miR-770-5p were forecasted using the online database MicroT-CDS. We selected TIMP3 for future research. TIMP3 maintains renal homeostasis, and TIMP3 depletion is a hallmark of diabetic nephropathy [34]. A previous study showed that TIMP3 knockdown resulted in significant changes in peroxisomal and mitochondrial fatty acids β-oxidation, suggesting that loss of TIMP3 might be more susceptible to DN [35]. Wang et al*.* revealed that TIMP3 protected kidneys from damage by regulating tubulointerstitial fibrosis and apoptosis [36]. In the present study, TIMP3 was overtly down-regulated in DN. Additionally, TIMP3 was negatively regulated by miR-770-5p in podocytes. TIMP3 restricted apoptosis and inflammation in HG-treated podocytes. Meanwhile, rescue experiments exhibited that knockdown of TIMP3 recuperated the effect of miR-770-5p on DN progression.

## Conclusion

In conclusion, miR-770-5p silencing restrained apoptosis and inflammation of HG-induced podocytes via targeting TIMP3, indicating that the novel molecular mechanism may provide a new approach for podocyte injury. However, further *in vivo* experiments in mouse models are essential for confirming our conclusions.

## Highlights

- MiR-770-5p was up-regulated, and TIMP3 was down-regulated in DN.
- Depletion of miR-770-5p alleviated HG-induced apoptosis and inflammation.
- TIMP3 was a target of miR-770-5p.
- MiR-770-5p modulated diabetic nephropathy progression by targeting TIMP3.

## Supplementary Material

Supplementary Figures S1-S2

## Data Availability

The analyzed data sets generated during the present study are available from the corresponding author on reasonable request.

## Abbreviations

Bax: Bcl-2 associated X
Bcl-2: B-cell lymphoma 2
DN: diabetic nephropathy
ECM: extracellular matrix
ELISA: enzyme-linked immunosorbent assay
EMT: epithelial-to-mesenchymal transition
HG: high glucose
miRNA: microRNA
qRT-PCR: quantitative real-time PCR
TGF-β: transforming growth factor-β
TIMP3: tissue inhibitors of metalloproteinase 3
