# Supplementary material for: MiR-770-5p facilitates podocyte apoptosis and inflammation in diabetic nephropathy by targeting TIMP3
Source: Biosci Rep. 2020 Apr 29;40(4):BSR20193653. doi: 10.1042/BSR20193653 (PMC7189364; doi:10.1042/BSR20193653)
Supplement: Supplementary Figures S1-S2 [file BSR-2019-3653_supp.pdf]

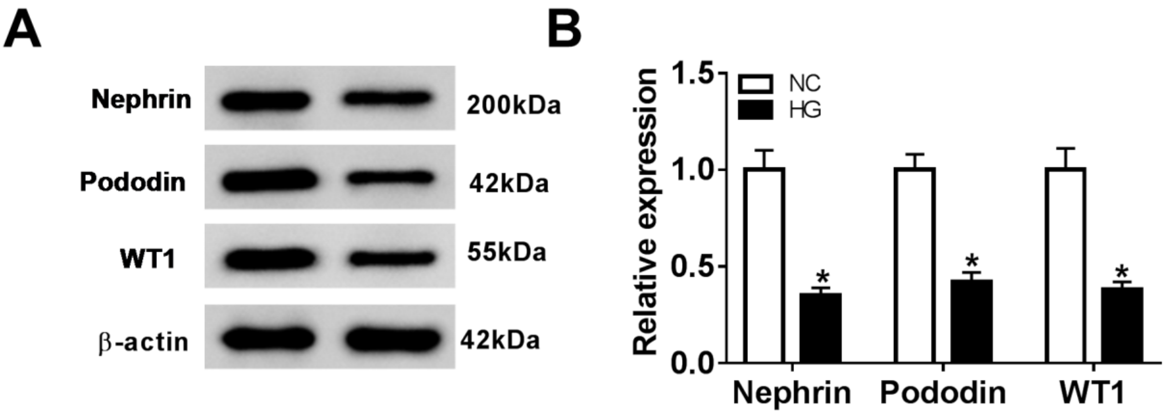
A
Nephrin
Pododin
WT1
β-actin
200kDa
42kDa
55kDa
42kDa
B
Relative expression
1.5
1.0
0.5
0.0
NC
HG
*
*
*
Nephrin
Pododin
WT1

Supplementary Figure S1 (A and B) Podocytes were exposed to 33 mM glucose for 24 h, andthe levels of nephrin, pododin and WT1 were measured by western blot assay.

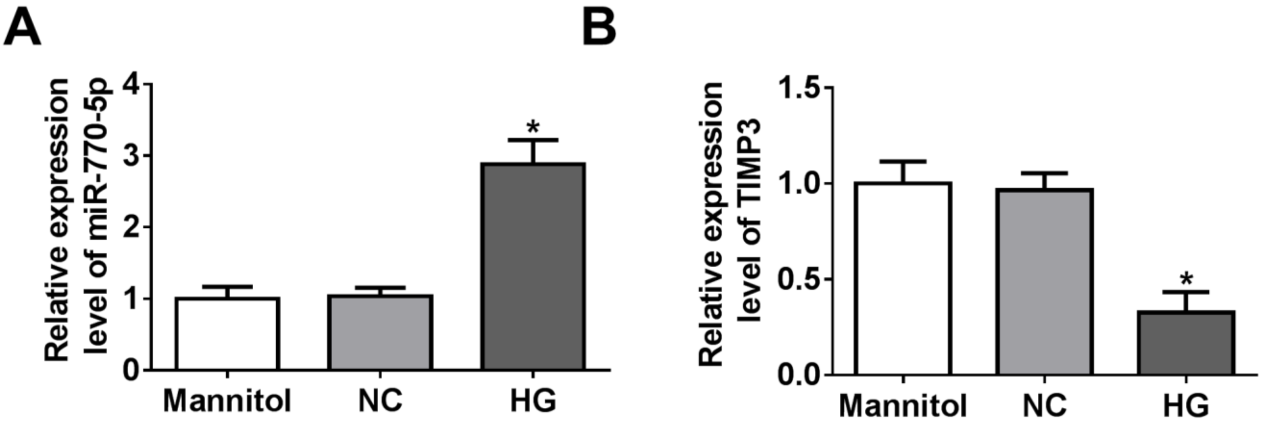
A
B
Relative expression level of miR-770-5p
Relative expression level of TIMP3
0
1
2
3
4
0.0
0.5
1.0
1.5
*
*
Mannitol
NC
HG
Mannitol
NC
HG

Supplementary Figure S2 (A and B) Podocytes were cultured under 33 mM high glucose (HG), 5.5 mM glucose (NC), or 5.5 mM glucose+27.5 mM mannitol (Mannitol) for 24 h, andthe levels of miR-770-5p and TIMP3 were measured by qRT-PCR.
